# Longitudinal analysis of total serum IgE levels with allergen sensitization and atopic diseases in early childhood

**DOI:** 10.1038/s41598-020-78272-8

**Published:** 2020-12-04

**Authors:** Chun-Ying Wong, Kuo-Wei Yeh, Jing-Long Huang, Kuan-Wen Su, Ming-Han Tsai, Man-Chin Hua, Sui-Ling Liao, Shen-Hao Lai, Li-Chen Chen, Chih-Yung Chiu

**Affiliations:** 1 College of Medicine, Chang Gung University, Taoyüan, Taiwan; 2 Division of Allergy, Asthma, and Rheumatology, Department of Pediatrics, Chang Gung Memorial Hospital and Chang Gung University College of Medicine, Taoyüan, Taiwan; 3 Department of Pediatrics, New Taipei Municipal TuCheng Hospital, Chang Gung Memorial Hospital and Chang Gung University, Taoyüan, Taiwan; 4 Department of Pediatrics, Chang Gung Memorial Hospital at Keelung, and Chang Gung University College of Medicine, Taoyüan, Taiwan; 5 Division of Pediatric Pulmonology, Chang Gung Memorial Hospital at Linkou, College of Medicine, Chang Gung University, Taoyüan, Taiwan

**Keywords:** Epidemiology, Paediatric research, Predictive markers, Asthma

## Abstract

There are few studies addressing the longitudinal analysis of serum IgE levels and its impact to the development of atopic diseases in early childhood. We investigated 170 children who regularly followed up at our clinic for 4 years in a birth cohort study with at least 3 time-points of serum samples. The pattern of total serum IgE levels from 6 months to 4 years of age was clustered using *K*-means method in R software. Specific immunoglobulin E antibodies against food (egg white and milk) and inhalant allergens (*D. pteronyssinus* and *D. farinae*) were measured at 0.5, 1, 1.5, 2, 3 and 4 years of age. By using *K*-means clustering, the dynamic changes in serum IgE levels was significantly stratified into 3 clusters (cluster A, < 100 kU/L, n = 106; cluster B, 100–200 kU/L, n = 35; cluster C, ≥ 200 kU/L, n = 29). A persistent total IgE levels higher than 100 kU/L appeared to be associated with higher prevalence of sensitization to food but not mite. However, a persistent IgE levels higher than 200 kU/L was not only remarkably related to increased prevalence of mite sensitization, but also risk of eczema at age 1 and allergic rhinitis and asthma at age 2, 3 and 4. In conclusion, a persistent total serum IgE level ≥ 200 kU/L since infancy is strongly associated with the presence of food and mite sensitization, as well as the development of eczema in infants, and rhinitis and asthma later in early childhood.

## Introduction

Immunoglobulin E (IgE) is widely known for its role in allergic reactions. IgE, produced by plasma cell, can recognize an allergen specifically and mediate an immune response. The immune system becomes sensitized, such that subsequent encounters with the same allergen lead to release of varies chemokines and cytokines, which results in causing symptoms of atopic diseases, for instance, local inflammation in eczema, mucous hypersecretion in rhinitis and bronchospasm in asthma^1^.

Several studies have presented the relationships between total serum IgE and allergen sensitization in children of different ages^2,3^. An increase in total serum IgE levels in infancy is associated with food sensitization, while elevated total serum IgE levels during early childhood correlate strongly with mite sensitization^4^. In addition, an additive effect on total serum IgE production is perceived when there is combined allergen sensitization^4^. However, the dynamic changes of total serum IgE levels relevant to allergen sensitization have not been well determined.

The association between allergen sensitization and atopic diseases have been well demonstrated^5,6^. Clinically, food sensitization appears to be associated with eczema, whereas mite sensitization is strongly related to rhinitis and asthma^7,8^. However, total serum IgE level is considered as a high sensitivity predictor of atopic diseases^9^. Elevated total serum IgE indicates high possibility of the presence of atopic diseases in children with allergy-like symptoms. Nevertheless, the longitudinal trends of total serum IgE levels and their association with allergen sensitization and atopic diseases during early childhood are still lacking.

The major aim of this study was to determine the total serum IgE levels from 6 months to 4 years of age in children from a Taiwan birth cohort study. The dynamic changes of total serum IgE levels were analyzed and their relevance to the presence of allergen sensitizations and risk for atopic diseases was also examined.

## Results

### Study population

In the birth cohort, initially 258 children were recruited, out of which, 182 (70.5%) children completed a 4-year follow-up at the clinic. A total of 170 children with serum samples obtained at least 3 time-points during the follow-up period were enrolled into this study. There were no significant differences in the baseline characteristics among these 170, 198, and all the 258 children studied^10^. At 4 years of age, atopic diseases including eczema, rhinitis, and asthma were physician-diagnosed in 20, 77, and 31 of these 170 children, respectively. The demographic characteristics of enrolled subjects, total serum IgE levels, and the diagnosis of atopic disease from 6 months to age 4 are summarized in Supplementary File S1.

### Clustering analysis of total serum IgE levels

**Figure 1:**
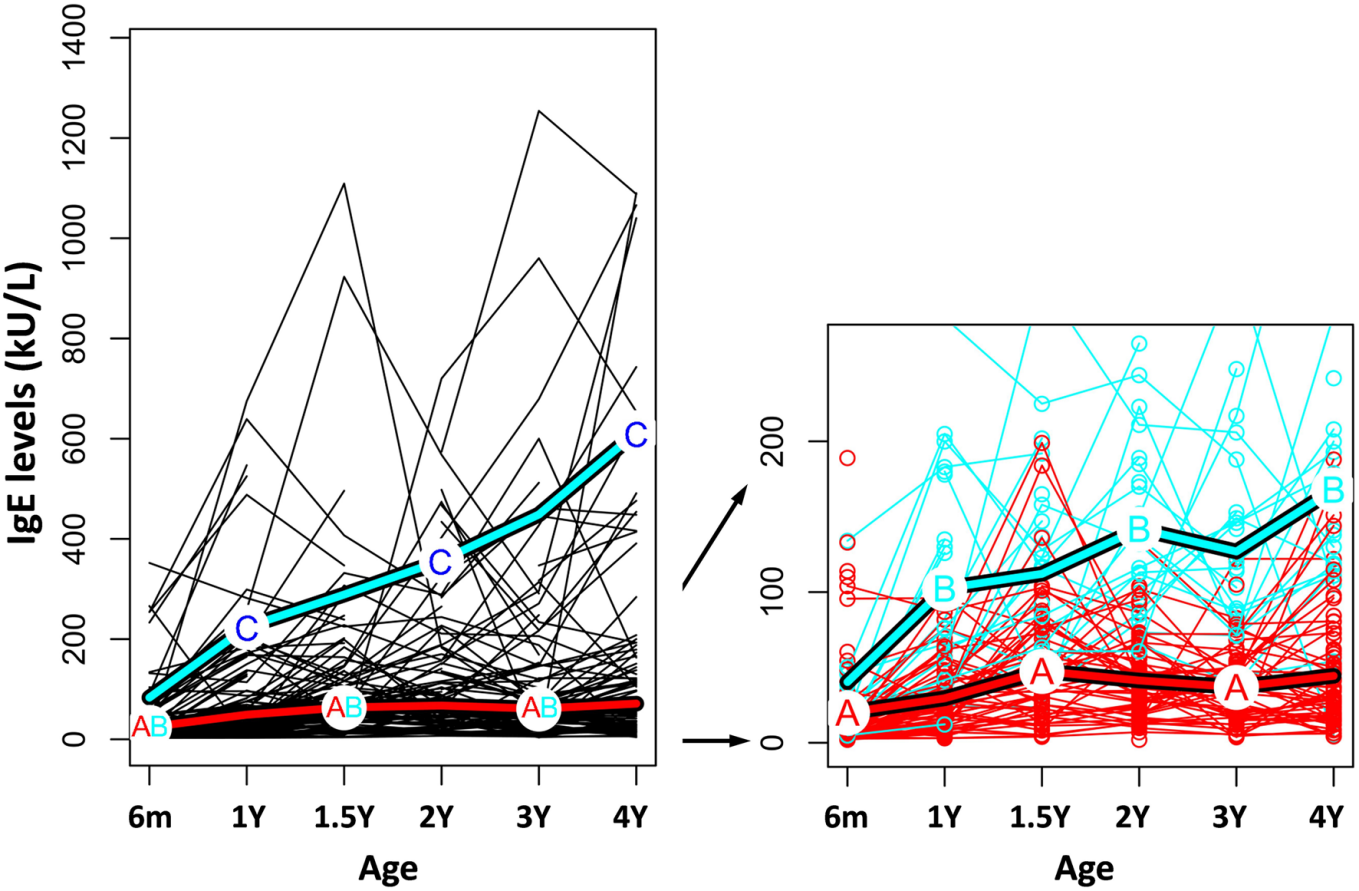
The pattern of total serum IgE levels from 6 month to age 4 clustering by using *K*-means method in R software. Cluster A (n = 106), IgE levels < 100 kU/L from birth to age 4, cluster B (n = 35), IgE levels 100–200 kU/L from age 1 to 4; cluster C (n = 29), IgE level ≥ 200 kU/L after age 1.

*K*-means clustering was performed for serum IgE levels from 6 months to 4 years of age using R software in 170 children. The dynamic changes in serum IgE levels during early childhood were stratified into three clusters notably (Fig. 1). Cluster A (n = 106) comprised children with serum IgE level persistently lower than 100 kU/L, throughout the 4-year-study period; cluster B (n = 35) comprised children with serum IgE level between 100 and 200 kU/L from age 1 to 4; and cluster C (n = 29) comprised children with increased IgE level ≥ 200 kU/L after age 1. Table 1 shows the baseline characteristics of 170 children in relation to clustering of total serum IgE levels. There were no significant differences in their characteristics among these three clusters.

**Table 1:** Baseline characteristics of 170 children in relation to total serum IgE clustering from 6 months to the age of 4 years.

Characteristics	Total serum IgE levels			
	< 100 kU/L (A, n = 106)	100–200 kU/L (B, n = 35)	≥ 200 kU/L (C, n = 29)	*P* value
**Family**				
Maternal atopy	46 (43.8%)	16 (45.7%)	10 (34.5%)	0.612
Paternal atopy	57 (54.3%)	20 (57.1%)	19 (65.5%)	0.557
Parental smoking	58 (54.7%)	21 (60.0%)	13 (44.8%)	0.470
Household income				0.213
Low, ≤ 500,000 NTD	41 (39.0%)	12 (34.3%)	12 (41.4%)	
Medium, 500,000–1,000,000 NTD	49 (46.7%)	12 (34.3%)	13 (44.8%)	
High, > 1,000,000 NTD	15 (14.3%)	11 (31.4%)	4 (13.8%)	
**Infant**				
Sex, male	56 (52.8%)	21 (60.0%)	21 (72.4%)	0.159
Maternal age (yr)	30.6 ± 4.6	31.2 ± 4.5	30.7 ± 4.2	0.798
Gestational age (wk)	37.9 ± 2.1	38.3 ± 1.6	38.5 ± 1.2	0.591
Birth BMI (kg/m^2^)	12.4 ± 1.6	12.7 ± 2.7	13.0 ± 3.2	0.955
Breastfeeding ≥ 6 months				0.354
Exclusive	39 (36.8%)	8 (22.9%)	12 (41.4%)	
Partial	38 (35.8%)	12 (34.3%)	8 (27.6%)	
Formula	29 (27.4%)	15 (42.9%)	9 (31.0%)	

Data shown are mean ± s.d. or number (%) of patients as appropriate. NTD, new Taiwan dollar; yr, year; wk, week; BMI, body mass index.

### Correlation between serum IgE levels and allergen sensitization

Table 2 summarizes the correlations of total serum IgE levels with allergen-specific IgE levels at different years of age. There was a significantly positive correlation between serum IgE levels and egg white- and milk-specific IgE levels at age 0.5, 1, 1.5 and 2. However, a significantly positive correlation was found between serum IgE levels and *D. farinae*- and *D. pteronyssinus-*specific IgE levels at 2, 3 and 4 years of age.

**Table 2:** Correlations of total serum IgE levels with allergen-specific IgE levels at different years of age.

Total IgE	Food sensitization				Mite sensitization			
	Egg white		Milk		*D. pteronyssinus*		*D. farinae*	
	r	*P*	r	*P*	r	*P*	r	*P*
Age 0.5	0.155	0.067	0.291	** < 0.001**	0.100	0.236	0.137	0.106
Age 1	0.320	** < 0.001**	0.507	** < 0.001**	0.083	0.336	0.078	0.356
Age 1.5	0.340	** < 0.001**	0.128	0.174	0.182	0.052	0.160	0.089
Age 2	0.473	** < 0.001**	0.313	**0.001**	0.604	** < 0.001**	0.566	** < 0.001**
Age 3	0.148	0.137	0.169	0.089	0.505	** < 0.001**	0.474	** < 0.001**
Age 4	0.298	**0.006**	0.161	0.141	0.664	** < 0.001**	0.651	** < 0.001**

The correlations between total serum IgE levels and allergen-specific IgE levels were conducted using Pearson’s rank correlation coefficient. All *P* values < 0.05, which is in bold, are significant.

### Association between serum IgE clusters and allergen sensitization

**Figure 2:**
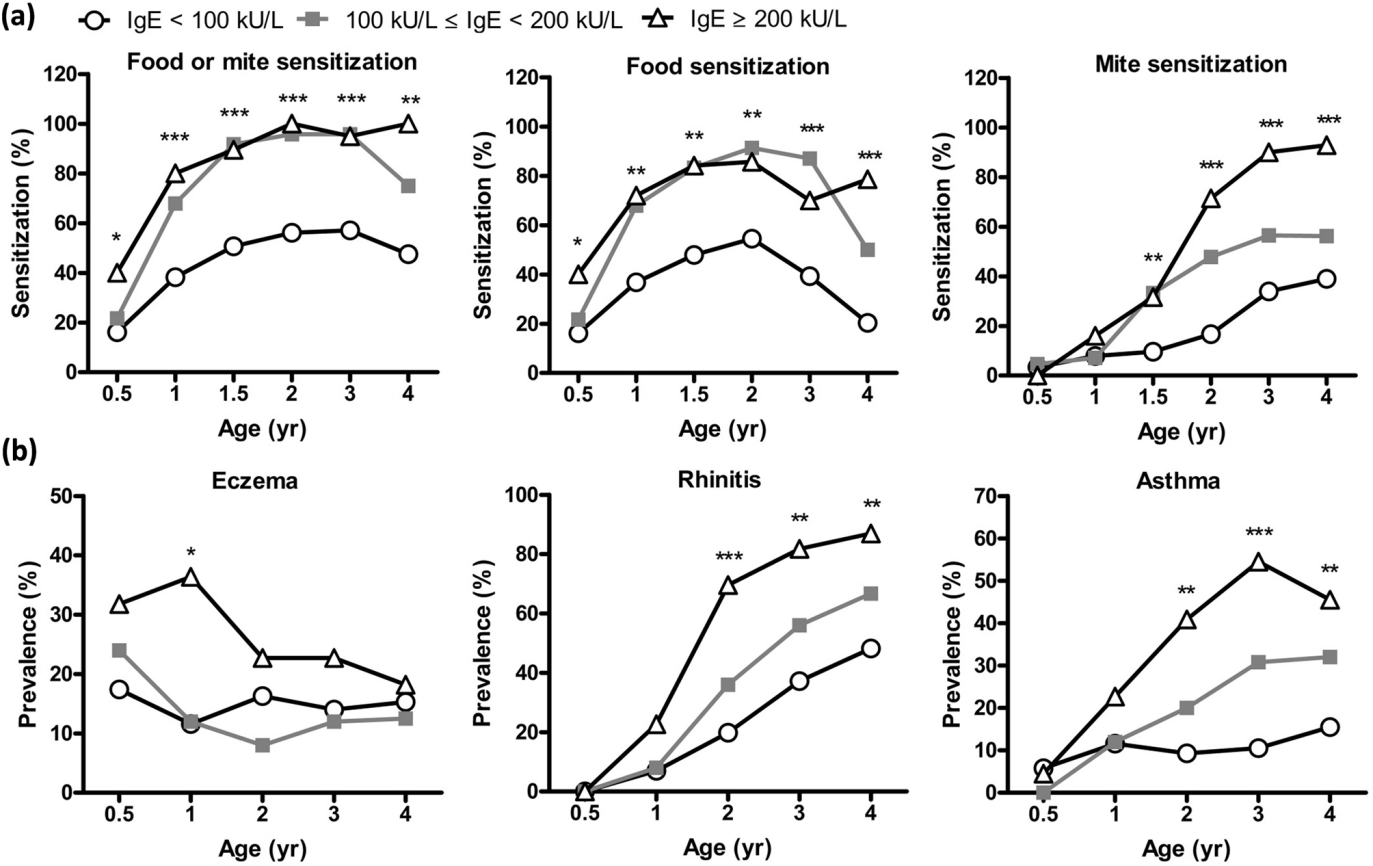
The relationships between total serum IgE levels clustering with allergen sensitization to food and mite (**a**), eczema, rhinitis and asthma (**b**) at different years of age. *P < 0.05; **P < 0.01; ***P < 0.001. The prevalence of food sensitization was significantly higher in children with total IgE levels ≥ 100 kU/L from 6 months to age 4, while a significantly higher prevalence of mite sensitization was only found in children with higher serum IgE levels ≥ 200 kU/L after age 1. In children with total IgE levels ≥ 200 kU/L, the prevalence of eczema was significantly higher at age 1, while the prevalence of rhinitis and asthma was significantly higher at the age of 2 to 4.

Comparisons and differences between the three clusters with respect to total serum IgE levels and allergen sensitization are shown in Fig. 2a. A significantly higher prevalence of food sensitization was found in children with IgE sensitization (> 100 kU/L, cluster B and C) compared to children grouped in cluster A at different years of age. By contrast, a significantly higher prevalence of mite sensitization was only found in children with higher serum IgE levels (≥ 200 kU/L, cluster C) in comparison with children grouped in cluster A.

### Association between serum IgE clusters and atopic diseases

Figure 2b shows the relationship between three serum IgE clusters and development of eczema, rhinitis and asthma at different ages. The prevalence of eczema decreased but allergic rhinitis and asthma increased markedly since age 2. Higher serum IgE levels (≥ 200 kU/L, cluster C) was significantly associated with higher prevalence of eczema at age 1, and allergic rhinitis and asthma at age 2, 3 and 4. After adjusting confounding factors, compared with serum IgE level < 100 kU/L in cluster A, higher serum IgE levels (≥ 200 kU/L) in cluster C appeared to show a significantly increased risk of allergic rhinitis [odds ratio (OR), 9.09; 95% confidence interval (CI), 2.20–37.53; *P* = 0.002] and asthma (OR, 5.94; 95% CI, 1.89–18.69; *P* = 0.002) at the age of 4 years.

## Discussion

Total serum IgE level is commonly elevated in patients with allergic diseases. It has been suggested to be utilized in predicting the development of allergic disorders. However, the dynamic changes and values of total serum IgE levels relevant to the development of sensitization to allergens and risk of atopic diseases remains unclear, especially in children. This study provides the respective values of total serum IgE levels that have significant association with sensitization to various allergens and atopic diseases during different stages of childhood.

IgE is secreted by class-switched B cell. The class-switch recombination of a B cell requires an antigen-dependent receptor-ligand binding interaction with an activated Th2 cell. This process usually takes place within secondary lymphoid tissues. However, the somatic hypermutation and class-switch recombination of B cells are rare in infants below the age of 6 months^11^. The secondary lymphoid organs are not completely mature in infants, reducing the likelihood of IgE class switching^12^. In this study, the serum IgE levels in 6 month-old infant are generally low (< 100 kU/L) which is in agreement with previous studies that showed rarity of IgE-producing cells at 6 months of age^13^. Therefore, the serum IgE level of infant below the age of 6 months may not be useful in predicting the development of any diseases.

The production of IgE in infant older than 6 months starts to rise after exposing to food allergens^14,15^. Food sensitization in infant should be noticed early due to its essential association with eczema, a common allergic skin disease with disrupted skin barrier^16,17^. Eczema could be exacerbated by food allergens by initiating the immune response^18,19^. To prevent eczema flare ups or from getting worse, early diagnosis and further avoidance of food allergens are relevant to infant. In our study, children with serum total IgE level > 100 kU/L are demonstrated to have food sensitization. A significantly higher prevalence of eczema was also found in children with serum total IgE level ≥ 200 kU/L at the age of 1 in our study. Therefore, total serum IgE level > 100 kU/L in infants may indicate they have food sensitization and a respectively higher risk of eczema. In addition, when infant has skin symptoms and with serum total IgE level ≥ 200 kU/L, physicians should consider the diagnosis of eczema, followed by early treatment and prevention.

The increase of serum total IgE level of children after the age of 2 is more related to mite sensitization^4^. Furthermore, children with high house dust mite-specific IgE level is at the highest risk of rhinitis and asthma^8,20^, which is in consonance with our study that an increased serum total IgE level (≥ 200 kU/L) strongly correlated with mite-specific IgE levels appeared to significantly increase risk of rhinitis and asthma after age 2. Thus, in clinical practice, young children with serum total IgE level ≥ 200 kU/L might have a high possibility of the presence of mite sensitization and rhinitis and asthma should be considered in such instances with allergic symptoms.

Limitations of this birth cohort include the relatively small enrolled population of 170 children, and thus limited statistical power to detect the association for subanalyses. However, the strength of this study is manifested by its long-term, longitudinal follow-up and regular measurement of total serum and allergen-specific IgE levels which established the dynamic relationships of IgE levels with allergen sensitizations and atopic disease development over time during early childhood.

In conclusion, serum total IgE level could be predictive of allergen sensitization and atopic diseases in early childhood. In infancy, serum total IgE level > 100 kU/L may reveal the presence of food sensitization, while serum total IgE level ≥ 200 kU/L may indicate a high risk of eczema. Young children with persistent serum total IgE level ≥ 200 kU/L appear to be associated with high prevalence of mite sensitization and be at risk for allergic rhinitis and asthma. Thus, for children with serum total IgE level ≥ 200 kU/L from infancy to early childhood, there is a high chance of developing eczema in infants and rhinitis and asthma later in life, providing early diagnosis and treatment for childhood atopic diseases. However, further studies with larger sample sizes are required to validate our findings.

## Methods

### Patients and data collection

We enrolled children who completed a 4-year follow-up in a birth cohort study launched in Taiwan. Detailed descriptions regarding subject recruitment of this birth cohort study were reported previously^4^. The detailed of information regarding demographic data, child’s sex, family history of atopy, exposure to passive smoking, household income, and history of breastfeeding was collected and analyzed. Atopic diseases were evaluated and diagnosed by the same pediatric pulmonologist at the clinic. Diagnosis of atopic diseases including eczema, allergic rhinitis, and asthma was described in our previous study^10,21^. This study was approved by the Ethics Committee of Chang Gung Memorial Hospital (No. 103-6236A3). All experiments in this study were performed in accordance with the relevant guidelines and regulations, and written informed consent was obtained from the parents or guardians of all the study subjects.

### Measurement of total serum IgE levels and clustering

Serum samples were collected and measured at 6 months, and 1, 1.5, 2, 3, and 4 years of age. As described in our previous study^10^, total serum IgE level was measured using ImmunoCAP (Phadia, Uppsala, Sweden) and IgE sensitization was defined as IgE levels > 100 kU/L. For clustering, total serum IgE levels at different ages were imported into R software (Version 3.6.3). *K*-means method was then used to group serum IgE levels into discrete and stable clusters of time series data from 6 months to 4 years of age.

### Measurement of allergen-specific IgE levels

Allergen-specific IgE was determined using a commercial assay for IgE (ImmunoCAP Phadiatop Infant; Phadia) for a mix of the two most common food allergens (egg white and milk) and inhalant allergens in Taiwan (*D. pteronyssinus* and *D. farinae*) as described previously^22,23^. Allergen sensitization was defined as values ≥ 0.35 kU/L^24^.

### Confounders

Confounding factors associated with atopic disease development, such as child’s gender, maternal and gestational age at birth, maternal atopy, elder siblings at birth, prenatal exposure to passive smoking, patterns of breastfeeding practices among infants, and family income, were collected and analyzed using multiple logistic regression analysis.

### Statistical analysis

*K*-means clustering of total serum IgE levels was calculated in R software. Univariate parametric and nonparametric tests such as ANOVA, χ^*2*^, Fisher’s exact test, and Kruskal–Wallis rank sum test were used to compare baseline characteristics and allergic sensitization among serum IgE level clusters. Pearson’s correlation test was used to determine the correlation between the total serum IgE and allergen specific IgE levels. Multiple logistic regression analysis was used to determine the association between serum IgE level clusters and atopic diseases by adjusting for confounders. The Statistical Package for the Social Sciences (SPSS Statistics for Windows Version 20.0; Armonk, NY, USA) software was used for statistical analysis of data, and GraphPad Prism software (GraphPad Software Inc. Version 5.01; San Diego, CA, USA) was used to represent data graphically. Statistical hypothesis tests were two-tailed with a significance level of 0.05.

## Supplementary information

Supplementary Information.

## Data Availability

The datasets generated during and/or analyzed during the current study are not publicly available duo to the personal privacy of subjects but are available from the corresponding author on reasonable request.
